# Supplementary material for: A positively selected microRNA controls a reversible aging program in striated muscle
Source: bioRxiv. 2026 Sep 7:2026.09.03.749250. Preprint. [Version 1] doi: 10.64898/2026.09.03.749250 (PMC13622596; doi:10.64898/2026.09.03.749250)
Supplement: Supplementary file 1 [file media-1.pdf]

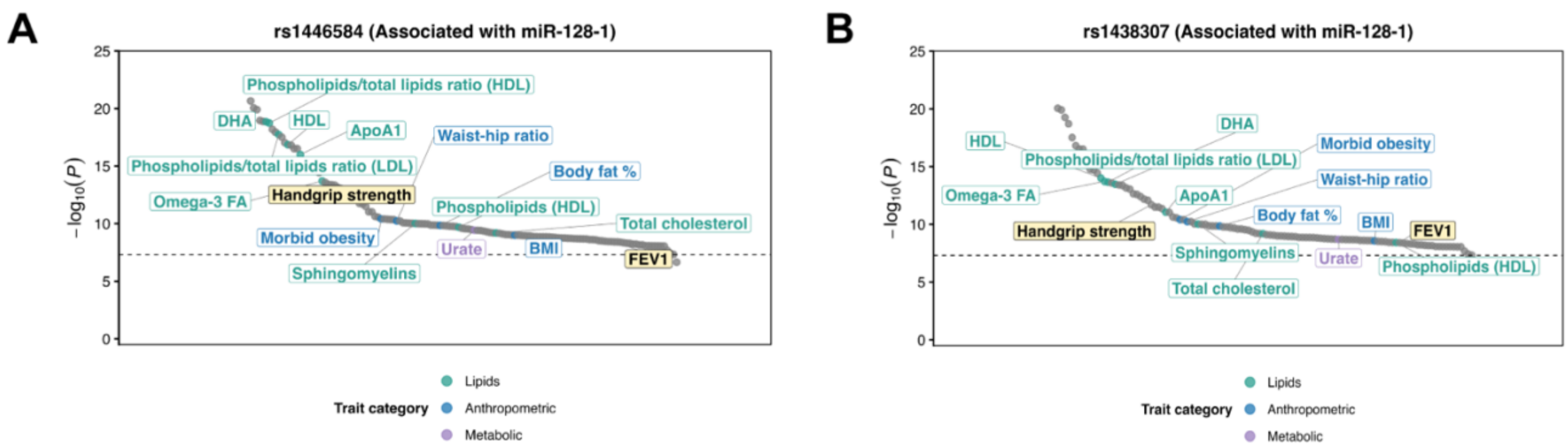

Figure S1. Additional SNPs in the miR-128-1 locus associate with muscle and pulmonary function in humans.

(A) PheWAS plot of the single nucleotide polymorphism (SNP) rs1446584 at the miR-128-1 locus. Dashed line denotes genome-wide significance ($P<5 \times 10^{-8}$).
(B) PheWAS plot of the single nucleotide polymorphism (SNP) rs1438307 at the miR-128-1 locus. Dashed line denotes genome-wide significance ($P<5 \times 10^{-8}$).

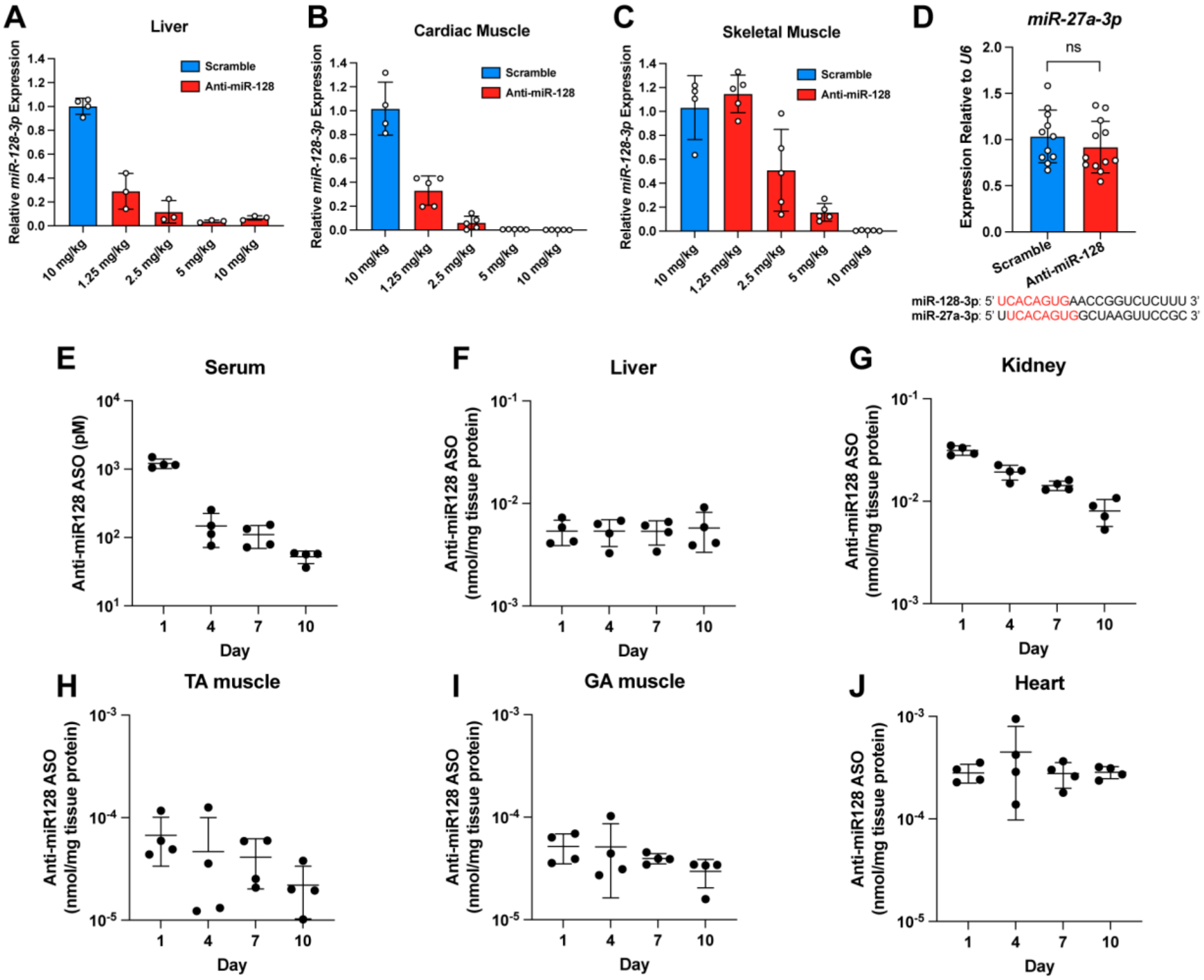

Figure S2. Dose response, specificity, and tissue kinetics of anti-miR-128.

(A-C) *miR-128-3p* expression levels measured 28 days after a single treatment of C57BL/6J mice with scramble (10 mg/kg) or anti-miR-128 (10, 5, 2.5, 1.25 mg/kg) in liver (A), cardiac muscle (B), or skeletal muscle (C).
(D) RT-qPCR analysis of non-target microRNA, miR-27a-3p following anti-miR-128 or scramble treatment in gastrocnemius muscle of 106-week-old C57BL/6J mice that had been treated for 20 weeks. The sequence comparison of miR-128-3p to miR-27a-3p is shown, with shared nucleotides highlighted in red.
(E-J) Anti-miR-128 concentrations measured by SplintR qPCR at 1, 4, 7, or 10 days after a single treatment at 10 mg/kg in C57BL/6J mice in serum (E), liver (F), kidney (G), TA muscle (H), GA muscle (I), and heart tissue (J).

Data are presented as mean +/- SD. N=4 per group (A-C, E-J) and N=11-12 per group (D). ns= not significant (unpaired two-tailed t-test (D)).

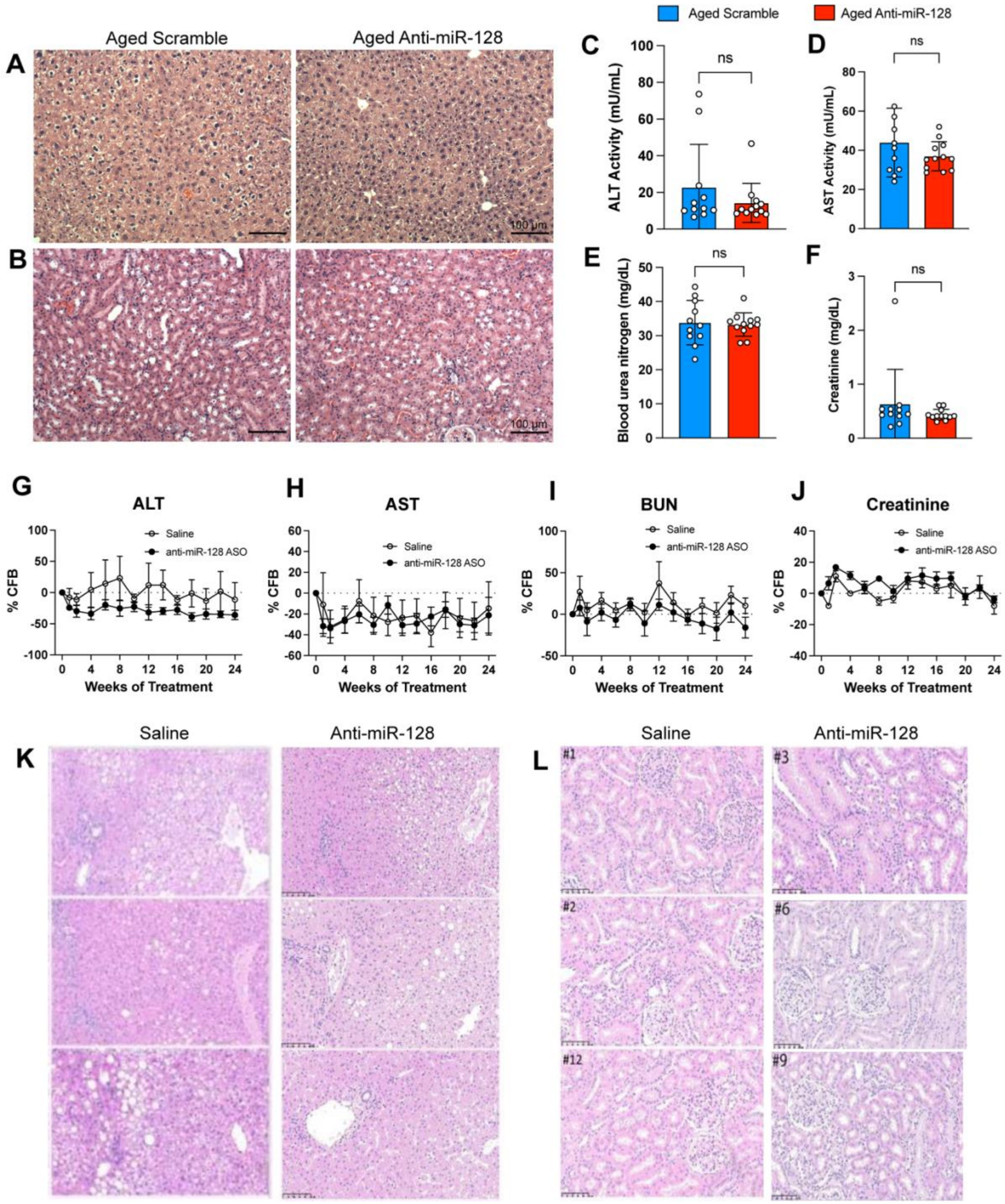

Figure S3. Cross-species safety profile of long-term anti-miR-128 treatment.

(A-F) Toxicity analysis of serum and tissue from 106-week-old C57BL/6J mice that had been treated with anti-miR-128 or scramble at 10 mg/kg for 20 weeks. H&E-stained liver (A) and kidney (B), scale bar, 100 μm (inset). Serum ALT activity (C), AST activity (D), blood urea nitrogen (E), and creatinine (F) measured at 20 weeks of treatment.

(G-L) Toxicity analysis of serum and tissue from cynomolgus monkeys treated with saline or anti-miR-128 at 5 mg/kg for 24 weeks. Serum ALT activity (G), AST activity (H), blood urea nitrogen (I), and creatinine (J) measured at 14 scheduled timepoints during treatment. H&E-stained liver (K) and kidney (L) tissue, scale is 100 μm (inset).

Data are presented as mean +/- SD. N=11-12 per group (mice); N=3-4 per group (monkeys) ns, not significant by unpaired, two-tailed, t-test (C-F).

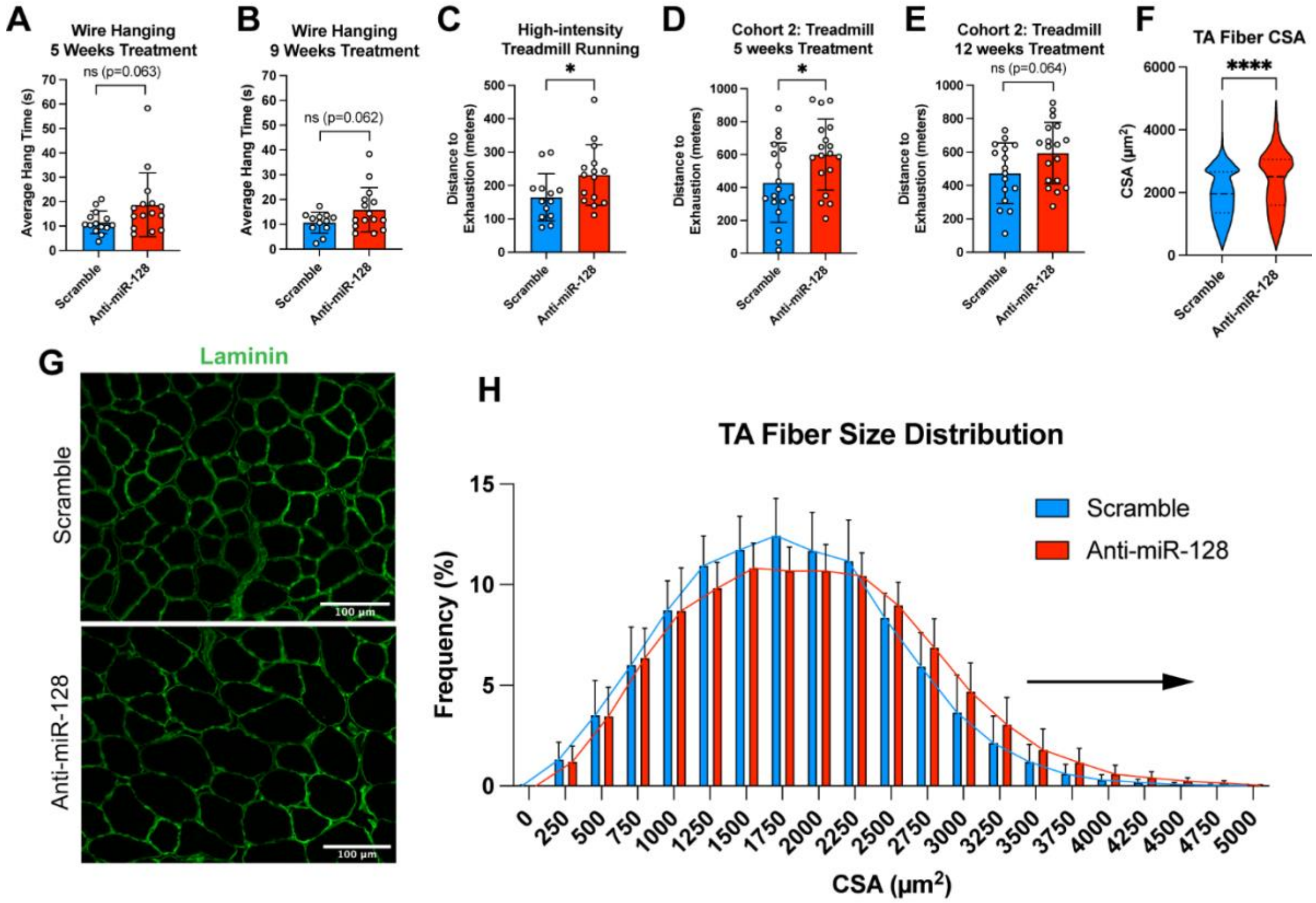

Figure S4. Additional functional and histological analyses in aged mice treated with anti-miR-128.

(A) Wire hanging at 5 weeks of treatment from cohort 1.
(B) Wire hanging at 9 weeks of treatment from cohort 1.
(C) Distance run to exhaustion using a high intensity treadmill protocol at 8 weeks of treatment from cohort 1.
(D) Distance run to exhaustion at 5 weeks of treatment from cohort 2.
(E) Distance run to exhaustion at 12 weeks of treatment from cohort 2.
(F-H) Muscle fiber cross sectional area (CSA) by laminin staining from TA muscle of scramble or anti-miR-128 treated mice from cohort 1. Fiber CSA by group (F). Representative immunofluorescence images of TA muscle stained for laminin (Scale bar, 100 μm; inset) (G). Frequency distribution of TA muscle fiber CSA quantified for each mouse and averaged by treatment group (H).

Data are presented as mean +/- SD. N=14-15 per group (A); N=12-15 per group (B); N= 13-15 per group (C); N=18 (D); N=16-18 (E); N=11 per group (H). ns, not significant, *p<0.05,**p<0.01,***p<0.001,****p<0.0001 (unpaired, two-tailed t-test (A-E), Kolmogorov-Smirnov test (F)).

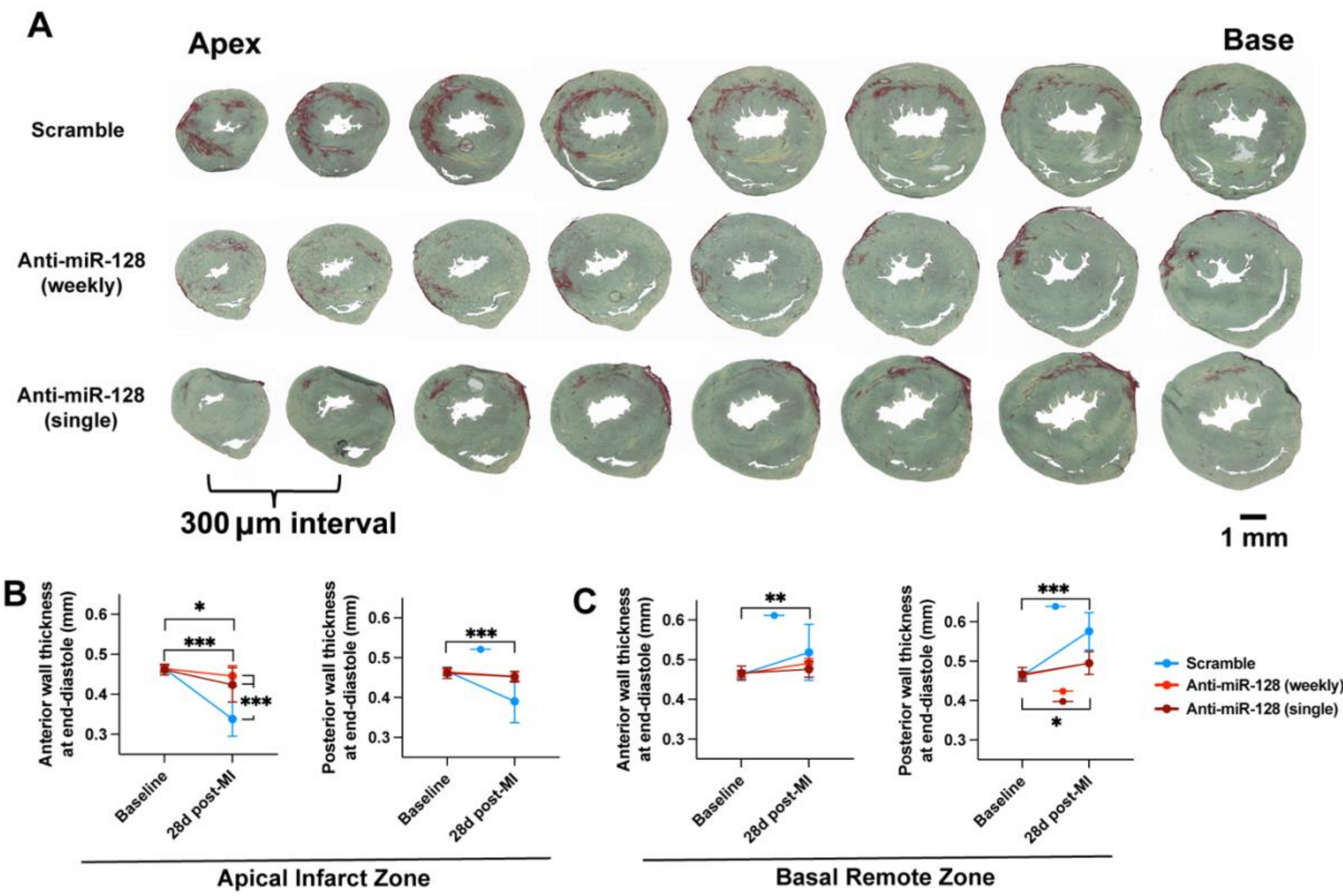

Figure S5. Additional fibrosis images and wall thickness measurements from post-I/R mice.

(A) Representative serial cryosections from the apex to base of the ventricle for infarct scar size measurement after I/R MI (cohort 1). Heart samples were sliced transversely at 10-µm thickness with an interval of 300 µm between each section. Eight cryosections from the apex to base of the ventricle were stained with Picrosirius Red and Fast Green for infarct scar size measurement. Mice were treated with scramble control ASO (weekly injections; top), or anti-miR-128 (weekly injections; middle), or anti-miR-128 (single injection; bottom).
(B-C) Anterior and posterior wall thickness by echocardiography at apical infarct zone (B) and basal remote zone (C) at baseline and 28 days post-I/R from cohort 1.

Data are presented as mean +/- SD. N=8-10 per group (B-C). *$p<0.05$, **$p<0.01$,***$p<0.001$,****$p<0.0001$ (two-way ANOVA followed by the Šidák method (B-C)).

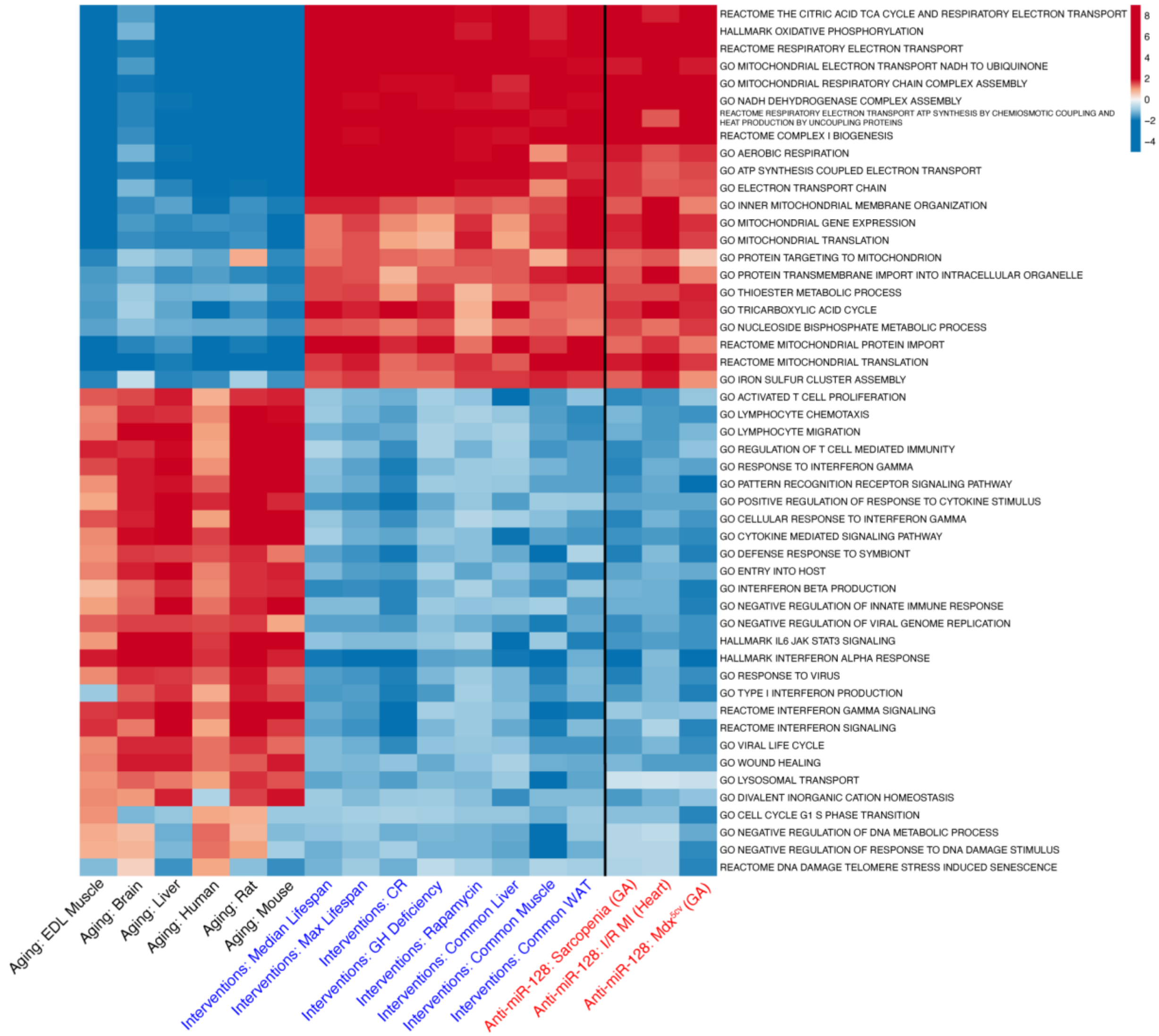

Figure S6. Expanded pathway analysis of mitochondrial and inflammatory transcriptional signatures rescued by anti-miR-128 across aging and disease contexts.

Gene set enrichment analysis of the expression profile of aged C57BL/6J gastrocnemius muscle, *mdx*^{5cv} gastrocnemius muscle, and post-I/R cardiac tissue treated with anti-miR-128 (red text) compared to signatures of mammalian aging (black text) and signatures of longevity interventions (blue text). Red boxes indicate upregulated pathways, and blue boxes indicate downregulated pathways. Additional pathways within the mitochondrial and inflammatory categories are shown beyond those depicted in Figure 7.

Supplementary Table 1. Serum Chemistry & Hematology — NHP Safety Study

Data are presented as mean ± SD. N = 3 (saline), 4 (anti-miR-128) per group. ns, not significant; *p<0.05, **p<0.01, ***p<0.001 (unpaired, two-tailed Welch's t-test vs. saline at the same timepoint). The primary analysis reported here is change from each animal's own week-0 value (see Results); week-0 comparisons precede the first dose and reflect baseline group differences, not treatment effects. Reference intervals have been omitted: those supplied with the source panels are generic adult-primate ranges rather than cynomolgus-specific, and several analytes fall wholly outside them in both arms at every timepoint, amylase most clearly (observed 92-248 U/L against a reported interval of 1,000-2,500 U/L). CPK, see footnote a; chloride, see footnote b.

Analyte	Units	Saline				Anti-miR-128			
		Week 0	Week 8	Week 16	Week 24	Week 0	Week 8	Week 16	Week 24
**HEPATIC FUNCTION**									
Total protein	g/dL	7.3 ± 0.5	7.3 ± 0.4	7.4 ± 0.3	7.8 ± 0.6	7.9 ± 0.2	7.7 ± 0.2	7.7 ± 0.2	8.0 ± 0.5
Albumin	g/dL	3.6 ± 0.2	3.7 ± 0.1	3.8 ± 0.1	3.8 ± 0.2	3.8 ± 0.1	3.8 ± 0.2	3.8 ± 0.1	3.7 ± 0.1
Globulin	g/dL	3.7 ± 0.3	3.6 ± 0.3	3.6 ± 0.2	4.0 ± 0.6	4.1 ± 0.2	3.9 ± 0.2	3.9 ± 0.2	4.3 ± 0.4
AST	U/L	61.0 ± 23.9	43.7 ± 15.0	34.0 ± 5.3	45.3 ± 15.0	43.2 ± 6.2	29.5 ± 4.8	34.2 ± 12.7	33.0 ± 13.3
ALT	U/L	64.0 ± 21.3	77.0 ± 37.6	56.0 ± 16.1	50.3 ± 18.8	31.2 ± 8.3	22.5 ± 5.9	22.8 ± 8.5	20.0 ± 6.8
Alkaline phosphatase	U/L	209.0 ± 67.5	182.7 ± 83.6	146.0 ± 58.6	158.0 ± 59.4	144.8 ± 33.5	138.0 ± 34.2	110.8 ± 26.4	125.8 ± 33.5
GGT	U/L	98.3 ± 24.6	94.3 ± 25.0	99.3 ± 28.2	91.3 ± 26.3	99.5 ± 34.6	96.5 ± 34.2	82.8 ± 27.0	81.5 ± 26.1
Total bilirubin	mg/dL	0.2 ± 0.1	0.1 ± 0.1	0.2 ± 0.1	0.2 ± 0.1	0.2 ± 0.1	0.1 ± 0.0	0.1 ± 0.0	0.1 ± 0.1
**RENAL FUNCTION**									
BUN	mg/dL	11.3 ± 2.1	12.7 ± 1.5	11.0 ± 1.0	12.3 ± 1.5	12.5 ± 1.9	13.8 ± 1.3	11.5 ± 0.6	10.2 ± 2.2
Creatinine	mg/dL	1.3 ± 0.3	1.2 ± 0.3	1.3 ± 0.3	1.2 ± 0.3	1.3 ± 0.2	1.5 ± 0.2	1.5 ± 0.2	1.3 ± 0.1
Phosphorus	mg/dL	3.9 ± 0.5	4.1 ± 0.7	3.8 ± 0.6	3.6 ± 0.4	4.8 ± 0.5	4.8 ± 0.3	4.3 ± 0.8	4.7 ± 0.6*
**METABOLIC**									
Glucose	mg/dL	52.0 ± 15.1	56.0 ± 20.7	57.0 ± 20.4	46.3 ± 14.4	53.0 ± 10.7	57.2 ± 9.2	54.0 ± 11.6	51.5 ± 7.7
Cholesterol	mg/dL	333.0 ± 113.2	304.0 ± 91.1	328.7 ± 50.4	367.0 ± 69.9	251.3 ± 117.8	239.0 ± 117.1	229.2 ± 95.6	212.3 ± 114.1
Triglycerides	mg/dL	72.7 ± 28.3	66.3 ± 25.5	62.7 ± 1.5	66.0 ± 13.1	55.2 ± 23.3	54.5 ± 19.7	56.2 ± 25.4	52.2 ± 14.8
Amylase	U/L	146.0 ± 76.1	141.0 ± 60.8	140.3 ± 64.0	158.3 ± 67.0	147.2 ± 25.2	133.8 ± 21.9	133.2 ± 29.8	136.8 ± 22.8
CPK	U/L	259.3 ± 67.4	564.3 ± 255.5	339.3 ± 233.5	338.3 ± 300.8	850.2 ± 841.8[a]	305.5 ± 214.8	1070.2 ± 1407.8[a]	263.2 ± 128.6
**ELECTROLYTES & MINERALS**									
Calcium	mg/dL	9.1 ± 0.1	9.2 ± 0.1	9.4 ± 0.1	9.7 ± 0.1	9.2 ± 0.1	9.0 ± 0.1	9.3 ± 0.1	9.5 ± 0.4
Magnesium	mEq/dL	1.3 ± 0.1	1.2 ± 0.0	1.2 ± 0.1	1.3 ± 0.1	1.4 ± 0.2	1.3 ± 0.0*	1.4 ± 0.0*	1.4 ± 0.1
Sodium	mEq/L	148.7 ± 1.2	147.0 ± 1.7	147.0 ± 1.7	146.0 ± 2.0	147.2 ± 3.3	145.8 ± 2.2	147.5 ± 1.9	147.5 ± 2.1
Potassium	mEq/L	4.0 ± 0.5	3.9 ± 0.1	3.7 ± 0.1	4.0 ± 0.6	3.7 ± 0.4	3.6 ± 0.2	3.6 ± 0.2	3.7 ± 0.1
Chloride	mEq/L	109.3 ± 0.6	107.0 ± 1.0	107.7 ± 0.6	115.7 ± 15.3[b]	106.0 ± 0.8**	105.8 ± 1.0	108.0 ± 2.6	106.8 ± 3.3
**HEMATOLOGY**									
RBC	$\times 10^6/\mu L$	6.3 ± 0.3	6.1 ± 0.5	6.2 ± 0.3	6.3 ± 0.5	6.9 ± 0.2*	6.4 ± 0.3	6.6 ± 0.2	6.8 ± 0.3
HGB	g/dL	13.0 ± 0.3	12.4 ± 0.7	12.9 ± 0.7	12.7 ± 0.4	13.8 ± 0.3*	13.1 ± 0.5	13.6 ± 0.1	13.4 ± 0.1
HCT	%	44.3 ± 0.6	41.3 ± 1.2	42.0 ± 2.6	43.3 ± 2.1	48.0 ± 0.8***	43.5 ± 1.7	45.2 ± 1.3	45.8 ± 1.3
MCV	fL	71.0 ± 2.6	67.7 ± 4.0	68.3 ± 4.6	68.3 ± 3.8	69.8 ± 2.5	67.8 ± 4.2	69.0 ± 2.4	67.5 ± 1.3
MCH	pg	20.8 ± 0.5	20.4 ± 0.4	20.7 ± 0.7	20.1 ± 1.1	20.1 ± 0.4	20.4 ± 1.0	20.5 ± 0.6	19.7 ± 0.6

MCHC	g/dL	29.3 ± 0.6	30.3 ± 1.5	30.7 ± 1.2	29.3 ± 0.6	29.0 ± 0.8	30.2 ± 0.5	29.8 ± 0.5	29.2 ± 0.5
WBC	×10³/µL	11.0 ± 1.7	11.1 ± 3.5	9.6 ± 2.4	10.6 ± 3.1	9.2 ± 0.6	8.5 ± 1.6	8.5 ± 2.1	9.5 ± 1.9
Platelet count	×10³/µL	367.7 ± 38.0	398.3 ± 31.8	407.0 ± 58.6	433.0 ± 44.4	291.3 ± 40.1	355.0 ± 80.2	379.8 ± 83.5	396.0 ± 133.8
Neutrophils	/µL	7432 ± 2036	8108 ± 3165	6725 ± 2365	6912 ± 2016	5743 ± 445	6352 ± 1782	5648 ± 2393	5951 ± 2199
Bands	/µL	0 ± 0	0 ± 0	0 ± 0	0 ± 0	0 ± 0	0 ± 0	0 ± 0	0 ± 0
Lymphocytes	/µL	2930 ± 892	2281 ± 158	2349 ± 219	2623 ± 482	2713 ± 702	1609 ± 268**	2260 ± 1152	2808 ± 1317
Monocytes	/µL	465 ± 85	538 ± 276	359 ± 65	933 ± 681	414 ± 60	272 ± 24	326 ± 18	443 ± 236
Eosinophils	/µL	172 ± 93	206 ± 78	167 ± 102	165 ± 224	330 ± 195	193 ± 150	176 ± 115	274 ± 212
Basophils	/µL	0 ± 0	0 ± 0	0 ± 0	0 ± 0	0 ± 0	0 ± 0	64 ± 129	0 ± 0

[a] Creatine kinase values in the anti-miR-128 arm at weeks 0 and 16 (850 ± 842 and 1,070 ± 1,408 U/L) are attributable to a single animal (UG228: 2,080 U/L pre-dose and 3,170 U/L at week 16), which returned to 402 U/L by week 24 on continued dosing. The remaining three treated animals ranged from 98 to 1,704 U/L across all timepoints. Transient creatine kinase elevation associated with restraint and ketamine sedation is well described in cynomolgus macaques.

[b] One saline animal (FR391) recorded a chloride of 133 mEq/L at week 24, against 105-109 mEq/L at its thirteen preceding draws and a concurrent sodium of 146 mEq/L. The value is reported as received from the analysing laboratory. The implied anion gap is not physiologically plausible and an analytical error is the most likely explanation; the value has been retained without correction and accounts for the elevated standard deviation shown.

Supplementary Table 2. Serum Chemistry — DMD Pig Study

Data are presented as mean ± SD. N = 3 per group. ns, not significant; *p<0.05, **p<0.01, ***p<0.001 $DMD^{Y/-}$ Scramble vs. WT (the Anti-miR-128 group was not compared with WT); °p<0.05 Anti-miR-128 vs. Scramble (unpaired, two-tailed Welch t-test within timepoint / Δ W8−W0).

Analyte	Units	Wild type (WT)[a]			$DMD^{Y/-}$ Scramble[a]			$DMD^{Y/-}$ Anti-miR-128[a]		
		Week 0	Week 8	Δ (W8 − W0) [%]	Week 0	Week 8	Δ (W8 − W0) [%]	Week 0	Week 8	Δ (W8 − W0) [%]
**MUSCLE DAMAGE**										
CK	10^3 U/L	1.18 ± 0.60	2.69 ± 1.68	267 ± 332	22.2 ± 11.7	103 ± 19.1*	518 ± 343	37.3 ± 30.5	67.8 ± 26.2	160 ± 127
ALAT	U/L	23.3 ± 3.5	38.3 ± 5.5	65.5 ± 19.1	679 ± 206*	511 ± 87.2*	-20.3 ± 21.0*	627 ± 214	487 ± 83.9	-19.0 ± 12.3
ASAT	U/L	41.7 ± 17.2	45.3 ± 9.7	19.2 ± 33.6	966 ± 717	840 ± 352	82.0 ± 184	1215 ± 742	1140 ± 420	16.6 ± 66.7
LDH	U/L	630 ± 87.4	603 ± 56.1	-3.6 ± 7.9	3971 ± 667*	8065 ± 2282*	108 ± 64.5	4956 ± 1191	12434 ± 5152	164 ± 112
**HEPATIC FUNCTION**										
ALP	U/L	481 ± 55.4	161 ± 19.1	-66.4 ± 4.1	165 ± 11.6**	114 ± 31.9	-31.5 ± 13.3*	154 ± 38.7	84.3 ± 9.7	-43.2 ± 9.8
Bilirubin, Total	µmol/L	5.0 ± 1.8	0.7 ± 0.3	-85.1 ± 6.5	2.0 ± 0.3	2.4 ± 1.0	14.4 ± 28.4*	1.8 ± 0.9	2.4 ± 0.5	66.6 ± 69.8
Total Protein	g/L	27.1 ± 2.1	57.0 ± 3.8	112 ± 19.9	27.1 ± 2.9	45.5 ± 3.4*	68.2 ± 7.0	23.1 ± 4.1	44.8 ± 2.3	98.3 ± 29.4
Albumin	g/L	23.6 ± 2.2	43.3 ± 1.2	84.4 ± 10.8	17.3 ± 2.8*	32.4 ± 2.8*	89.0 ± 13.1	14.0 ± 3.0	32.3 ± 1.8	137 ± 34.6
GGT	U/L	15.7 ± 4.0	24.7 ± 12.4	52.2 ± 31.5	27.3 ± 6.5	15.7 ± 2.9	-38.8 ± 21.2*	35 ± 16.8	22.3 ± 2.5°	-27.4 ± 22.6
Amylase	U/L	1553 ± 51.7	2218 ± 38.4	42.9 ± 5.6	1726 ± 178	1973 ± 403	13.6 ± 10.0*	1452 ± 240	1917 ± 502	33.2 ± 25.5
**RENAL FUNCTION**										
BUN	mmol/L	1.1 ± 0.0	3.5 ± 0.1	235 ± 13.3	0.6 ± 0.3	2.8 ± 1.7	492 ± 422	1.2 ± 0.4	2.6 ± 1.0	115 ± 20.9
Creatinine	µmol/L	48.7 ± 2.1	97.7 ± 1.5	101 ± 5.9	22 ± 3.5***	30.3 ± 12.7*	34.4 ± 27.7*	24 ± 7.0	35.3 ± 12.7	70.4 ± 97.4
**METABOLIC**										
Glucose	mmol/L	5.5 ± 0.3	7.4 ± 0.4	35.6 ± 9.4	6.4 ± 1.1	6.0 ± 0.9	-2.7 ± 28.3	5.2 ± 0.8	7.7 ± 3.2	54.4 ± 61.7
Cholesterol	mmol/L	2.9 ± 0.3	2.7 ± 0.2	-6.4 ± 2.6	1.2 ± 0.3**	1.7 ± 0.4*	40.0 ± 9.8*	1.2 ± 0.4	1.8 ± 0.2	67.7 ± 49.9
Triglycerides	mmol/L	0.8 ± 0.5	0.9 ± 0.2	49.3 ± 103	0.5 ± 0.1	0.8 ± 0.2	51.3 ± 46.1	0.6 ± 0.2	0.9 ± 0.3	68.8 ± 48.5
Fructosamine	µmol/L	131 ± 8.7	209 ± 5.7	59.2 ± 5.0	134 ± 3.5	180 ± 11.1*	34.9 ± 5.8*	124 ± 22.2	181 ± 11.9	50.5 ± 30.6
Lactate	mmol/L	2.3 ± 0.4	2.1 ± 0.1	-9.2 ± 10.0	2.7 ± 0.9	3.9 ± 0.6*	57.0 ± 53.0	1.5 ± 0.1	3.2 ± 0.8	112 ± 44.5
Lipase	U/L	6.5 ± 0.8	8.5 ± 0.8	31.6 ± 10.2	4.7 ± 0.7*	5.3 ± 0.4*	14.2 ± 6.2	4.3 ± 0.7	5.8 ± 0.3	38.5 ± 21.9
**ELECTROLYTES & MINERALS[b]**										
Phosphate	mmol/L	2.2 ± 0.3	3.2 ± 0.4	44.4 ± 9.1	2.2 ± 0.2	3.4 ± 0.3	53.8 ± 12.8	2.0 ± 0.4	3.5 ± 0.3	72.7 ± 15.5
Calcium	mmol/L	2.1 ± 0.0	2.8 ± 0.2	31.9 ± 6.8	2.3 ± 0.1	2.3 ± 0.1*	1.4 ± 3.1*	2.1 ± 0.3	2.4 ± 0.2	19.0 ± 8.8
Magnesium	mmol/L	0.8 ± 0.1	1.0 ± 0.1	37.1 ± 15.7	0.9 ± 0.1	0.9 ± 0.1	0.1 ± 13.0*	0.8 ± 0.1	0.9 ± 0.0	14.1 ± 13.4

[a] WT and $DMD^{Y/-}$ pigs in the different treatment groups were littermates. Reference ranges for pigs are not given as published porcine intervals vary by breed and age.

[b] Sodium, chloride, and potassium concentrations were consistently below the porcine reference ranges, indicating a possible analytical or preanalytical issue. These values were therefore excluded from further consideration.

Supplementary Table 3. RT-qPCR Primers.

Primer sequences used for RT-qPCR gene expression analysis.

Gene Symbol	Species	Forward Oligo (5'→3')	Reverse Oligo (5'→3')
Ppargc1a	Mouse	TATGGAGTGACATAGAGTGTGCT	CCACTTCAATCCACCCAGAAAG
Tfam	Mouse	TGGCAGTCCATAGGCACCGTATT	ACAGACAAGACTGATAGACGAGGG
Atp5g	Mouse	CCTGTGCCTGTCTTTCTACC	CCTTCCACACTCTGCCTTATC
Sod2	Mouse	CAGGATGCCGCTCCGTTAT	TGAGGTTTACACGACCGCTG
Ppara	Mouse	GTGCAGCCTCAGCCAAGTT	TGGGGAGAGAGGACAGATGG
Drp1	Mouse	GTTCCACGCCAACAGAATAC	CCTAACCCCCTGAATGAAGT
Fis1	Mouse	AAGTATGTGCGAGGGCTGT	TGCCTACCAGTCCATCTTTC
Mfn1	Mouse	GACCCGTGCGAAAGAGAGAG	TCGAGCAAAAGTAGTGGCCA
Mfn2	Mouse	ATGTTACCACGGAGCTGGAC	AACTGCTTCTCCGTCTGCAT
Opa1	Mouse	ATACTGGGATCTGCTGTTGG	AAGTCAGGCACAATCCACTT
R18s	Mouse	GCAATTATTCCCCATGAACG	GGCCTCACTAAACCATCCAA
Tnfa	Mouse	CCCTCACACTCAGATCATCTTCT	GCTACGACGTGGGCTACAG
Prkaa2	Mouse	GGAGAACACCAATTGACAGGC	GTGCTGATCACCTGGTAGAGT
Sirt1	Mouse	CGGCTACCGAGGTCCATATAC	CGCTTTGGTGGTTCTGAAAGG
Mt-co3	Mouse	AAATTCCTGTTGGAGGTCAGCAGC	TCGTACCACAGGCATTGTGATGGA
Actb	Mouse	TGTGACTTTGTGGTGTGGCTG	CACCTGCACTCTGGGTAAGGA

Supplementary Table 4. Trait Label Mapping for GWAS Credible Set Analysis.

Mapping of GWAS Catalog reported trait strings to simplified labels and broad trait categories used throughout the text and figures. Traits are grouped by category.

Reported Trait (GWAS Catalog)	Label (this study)
**Anthropometric**	
Body mass index	BMI
Obesity	Obesity
Obesity due to excess calories	Obesity
Morbid obesity (PheCode 278.11)	Morbid obesity
Body fat percentage (UKB data field 23099)	Body fat %
Waist-hip ratio	Waist-hip ratio
Body mass index (BMI) (UKB data field 21001)	BMI
Body mass index (BMI) (UKB data field 23104)	BMI
Body mass index (UKB data field 21001)	BMI
Arm fat mass left (UKB data field 23124)	Arm fat mass
Arm fat mass right (UKB data field 23120)	Arm fat mass
Arm fat percentage left (UKB data field 23123)	Arm fat %
Arm fat percentage right (UKB data field 23119)	Arm fat %
Waist circumference (UKB data field 48)	Waist circumference
Whole body fat mass (UKB data field 23100)	Whole body fat mass
Trunk fat percentage (UKB data field 23127)	Trunk fat %
Hip circumference (UKB data field 49)	Hip circumference
**Lipids**	
HDL cholesterol	HDL
HDL cholesterol levels	HDL
HDL cholesterol levels (UKB data field 30760)	HDL
High density lipoprotein cholesterol levels	HDL
High density lipoprotein cholesterol levels (UKB data field 30760)	HDL
High-density lipoprotein levels (MTAG)	HDL
Low density lipoprotein cholesterol levels	LDL
LDL direct levels (UKB data field 30780)	LDL
Cholesterol levels	Total cholesterol
Cholesterol levels (UKB data field 30690)	Total cholesterol
Total cholesterol levels	Total cholesterol
Total esterified cholesterol levels	Total cholesterol
Non-HDL cholesterol levels	Non-HDL cholesterol
Apolipoprotein B levels	ApoB
Apolipoprotein B levels (UKB data field 30640)	ApoB
Apolipoprotein A1 levels	ApoA1
Phospholipids in large HDL	Phospholipids (HDL)
Phospholipids in very large HDL	Phospholipids (HDL)
Phospholipids to Total Lipids in Large HDL percentage	Phospholipids/total lipids ratio (HDL)
Phospholipids to Total Lipids in Medium HDL percentage	Phospholipids/total lipids ratio (HDL)
Phospholipids to Total Lipids in Medium HDL percentage (PGS-adjusted)	Phospholipids/total lipids ratio (HDL)
Phospholipids to total lipids ratio in medium HDL	Phospholipids/total lipids ratio (HDL)

Phospholipids to Total Lipids in Large LDL percentage	Phospholipids/total lipids ratio (LDL)
Phospholipids to Total Lipids in Large LDL percentage (PGS-adjusted)	Phospholipids/total lipids ratio (LDL)
Omega-3 fatty acids	Omega-3 FA
Omega-3 fatty acids levels	Omega-3 FA
Omega-3 fatty acid levels	Omega-3 FA
Docosahexaenoic acid levels	DHA
Circulating docosahexaenoic acid levels	DHA
Sphingomyelins levels	Sphingomyelins
**Metabolic**	
Urate levels	Urate
Urate levels (UKB data field 30880)	Urate
Frailty (Factor 4 - Metabolic Problems)	Frailty (metabolic)
Polyneuropathy in diabetes (PheCode 250.6)	Diabetic polyneuropathy
**Cardiovascular event**	
Sudden cardiac arrest in coronary artery disease	Cardiac arrest (in CAD)
